# Inhibition of glial glutamate transporter GLT1 in the nucleus of the solitary tract attenuates baroreflex control of sympathetic nerve activity and heart rate

**DOI:** 10.14814/phy2.13877

**Published:** 2018-09-19

**Authors:** Kenta Yamamoto, Steve Mifflin

**Affiliations:** ^1^ Department of Physiology and Anatomy University of North Texas Health Science Centre Fort Worth Texas; ^2^ Faculty of Pharmaceutical Sciences Teikyo Heisei University Tokyo Japan

**Keywords:** Astrocytes, baroreflex, glutamate transporter, NTS

## Abstract

The astrocytic glutamate transporter (GLT1) plays an important role in the maintenance of extracellular glutamate concentration below neurotoxic levels in brain. However, the functional role of GLT1 within the nucleus of the solitary tract (NTS) in the regulation of cardiovascular function remains unclear. We examined the effect of inhibiting GLT1 in the subpostremal NTS on mean arterial pressure (MAP), renal sympathetic nerve activity (RSNA) and heart rate (HR) in anesthetized, artificially ventilated rats. It was found that dihydrokainate (DHK; inhibitor of GLT1, 5 mmol/L, 100 nL) injections into the NTS (*n* = 6) decreased MAP (50 ± 10 mmHg, mean ± SD), RSNA (89 ± 14%) and HR (37 ± 6 bpm). Pretreatment with kynurenate (KYN; glutamate receptor antagonist, 5 mmol/L, 30 *μ*L) topically applied to the dorsal surface of the brainstem (*n* = 4) attenuated the responses to NTS injections of DHK (*P* < 0.01). The effect of DHK on arterial baroreflex function was examined using i.v. infusions of phenylephrine and nitroprusside. DHK reduced baroreflex response range (maximum−minimum) of RSNA by 91 ± 2% and HR by 83 ± 5% (*n* = 6, *P* < 0.001). These results indicate that inhibition of GLT1 within the NTS decreases MAP, RSNA, and HR by the activation of ionotropic glutamate receptors. As a result, baroreflex control of RSNA and HR was dramatically attenuated. The astrocytic glutamate transporter in the NTS plays an important role in the maintenance and regulation of cardiovascular function.

## Introduction

Astrocytes are the most abundant type of glial cell in the central nervous system and they possess a glutamate transporter that removes neuronally released glutamate from the extracellular space. An important function of astrocytes is the maintenance of extracellular glutamate below neurotoxic levels in brain (Rothstein et al. 1996; Rao et al. 2001). Because glutamate is the principal excitatory neurotransmitter in brain, a second important function of the glutamate transporter on astrocytes is the termination of the action of the glutamate released by neurons (Anderson and Swanson 2000; Bunch et al. 2009), suggesting that the glutamate transporter on astrocytes could play a role in the regulation of the glutamatergic synaptic transmission (Huda et al. 2013). Since all peripheral afferent inputs to the nucleus of the solitary tract (NTS) appear to release glutamate as their primary neurotransmitter (Lawrence and Jarrott 1996), astrocytes in NTS may be critical for cardiovascular reflex control (Lin et al. 2013; Talman et al. 2017). In NTS, the main glutamate transporter expressed by astrocytes is astrocytic glutamate transporter 1 (GLT1) (Chounlamountry and Kessler 2011). However, recent studies have provided conflicting results about the role of astrocytic glutamate transporters in NTS in the regulation of cardiovascular function (Accorsi‐Mendonca et al. 2013; Costa et al. 2013; Matott et al. 2016, 2017). These conflicting results may be related to the fact that some studies examined overall astrocyte inhibition (Accorsi‐Mendonca et al. 2013; Costa et al. 2013) while others examined transporter function (Matott et al. 2016, 2017).

Matott et al. (2016) reported that glutamate transporters tonically restrain NTS excitability to modulate cardiorespiratory function. The NTS is the first site where baroreceptor inputs terminate within the central nervous system (Mifflin and Felder 1990). Inhibition of GLT1 in NTS resulted in responses consistent with baroreflex activation (e.g., reduced arterial pressure (AP), sympathetic nerve activity (SNA), and heart rate (HR), indicating that elevated levels of glutamate result in sustained activation of NTS neurons (Matott et al. 2017). Accordingly, we hypothesized that inhibition of GLT1 in NTS would attenuate baroreflex control of SNA and HR estimated by the response ranges of SNA and HR (i.e., the difference between maximum and minimum SNA and HR during baroreceptor loading and unloading). To test this hypothesis, we examined baroreflex stimulus‐response curves of renal SNA (RSNA) and HR during inhibition of GLT1 in the NTS.

## Materials and Methods

The Institutional Animal Care and Use Committee of the University of North Texas Health Science Center approved all experimental procedures.

### Surgical procedures

Experiments were performed on 19 male adult Sprague‐Dawley rats (430–560 g). All rats were given at least 1 week to acclimate to the holding facility before being used for any procedures. The rats were anesthetized with the long‐acting rodent anesthetic thiobutabarbital sodium (Inactin, SIGMA) at the initial dose of 110 mg/kg ip. Supplemental anesthetic was given in doses of 10 mg/kg ip as required to maintain a surgical plane of anesthesia (i.e., the absence of withdrawal to pinch of the hind paw and no evidence of fluctuations in AP in response to surgical manipulation or pinch of the hind paw following paralysis). Gallamine triethiodide (5 mg/kg/h) was infused intravenously to induce paralysis. Body temperature was maintained at approximately 37°C using a heating pad. The animals were intubated through a tracheotomy and mechanically ventilated with oxygen‐enriched room air. A catheter was inserted into the abdominal aorta via the femoral artery and was used for the measurement of AP. HR was calculated from the AP waveform. Catheters were inserted into the femoral vein for the infusion of drugs.

We exposed the left renal sympathetic nerve and attached a pair of Teflon‐coated stainless steel wires (AS632, Cooner Wire, CA, USA) to record RSNA. The nerve and electrodes were secured with silicone glue (Kwik‐Sil, World Precision Instruments, FL, USA). The nerve signals were amplified with band‐pass filters set between 100 and 1000 Hz. The filtered signals were full‐wave rectified and integrated using 50‐msec time constants to quantify RSNA.

### Microinjections into the NTS

The animal was placed in a stereotaxic frame and the dorsal surface of the brainstem was exposed. To determine the effect of inhibiting glutamate transporters in NTS in arterial baroreflex pathway, artificial cerebrospinal fluid (aCSF), dihydrokainate (DHK, 5 mmol/L), or DL‐threo‐*β*‐Benzyloxyaspartate (TBOA, 500 *μ*mol/L) was unilaterally injected using a glass micropipette into the subpostremal NTS (coordinates in mm with respect to calamus scriptorius: 0.5 rostral, 0.5 lateral, and 0.5 ventral from surface) (Sved and Tsukamoto 1992; Durgam et al. 1999). Microinjection volumes (100 nL) were measured by observing the movement of the fluid in a calibrated pipette (Calibrated Pipets 1–5 *μ*L, Drummond Scientific Company, PA, USA). DHK is selective inhibitor for GLT1 (Arriza et al. 1994; Wang et al. 1998). TBOA is a potent blocker of all subtypes of glutamate transporters in astrocytes and neurons. The concentrations and volumes of DHK and TBOA were determined based on preliminary experiments. Microinjection into NTS at concentration of DHK (0.5 mmol/L) and TBOA (50 *μ*mol/L) did not evoke AP response. To confirm the injection sites, 5% red or green latex microspheres (Lumiphore, CA, USA) were contained in the drugs.

### Protocols and data analysis

All experimental protocols were initiated at least 1 h after completion of the surgical procedures. We recorded AP, RSNA, and HR at a sampling rate of 1000 Hz. Because the absolute magnitude of RSNA depended on recording conditions, RSNA was presented as a percentage (%) of baseline. An intravenous bolus injection of the ganglionic blocker hexamethonium bromide (60 mg/kg) was used to determine “0%” RSNA. Baseline RSNA was used to determine “100%” RSNA.

#### DHK and TBOA microinjections into subpostremal NTS

**Figure 1:**
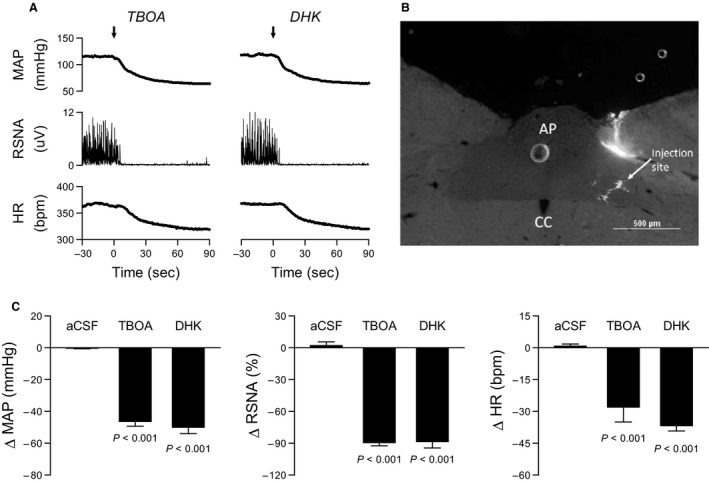
Inhibition of astrocytic glutamate transporter GLT‐1 in the subpostremal nucleus tractus solitarii (NTS) decreased mean arterial pressure (MAP), renal sympathetic nerve activity (RSNA), and heart rate (HR). (A) Typical recordings of MAP, RSNA, and HR in response to the TBOA (inhibitor for all subtypes of glutamate transporters) and DHK (selective inhibitor for glutamate transporter GLT1) injections. Arrows represent point of TBOA and DHK injections. (B) Typical injection site in subpostremal NTS. CC, central canal; AP, area postrema. (C) The TBOA and DHK injections decreased MAP, RSNA and HR compared to control injections of artificial cerebrospinal fluid (aCSF). *P* values compared with aCSF.

Baseline levels of mean AP (MAP), RSNA, and HR were obtained for 30 sec before aCSF (*n* = 6), TBOA (*n* = 6), or DHK (*n* = 6) microinjections. The injections were made using pressure over a period of 5–10 sec. The microinjection responses were measured between 60 and 90 sec after the microinjections (Fig. 1A). ΔMAP, ΔRSNA, and ΔHR were calculated as the difference in these values.

#### DHK microinjection into subpostremal NTS with glutamate receptor antagonist

DHK was injected into the NTS before, during, and after (recovery) topical application of the non‐selective ionotropic glutamate receptor antagonist kynurenate (KYN, 5 mmol/L, 30 *μ*L) on the exposed dorsal surface of the brainstem (*n* = 4) (Gourine et al. 2008). The interval between each injection was at least 30 min. ΔMAP, ΔRSNA, and ΔHR were calculated as described above.

#### Baroreflex curves during DHK microinjections

Recovery of MAP, RSNA, and HR responses to the DHK bolus injection began within 5 min which was too short a period to construct baroreflex curves. Therefore we examined the time course of MAP, RSNA, and HR responses to repeat unilateral injections of DHK at the subpostremal NTS (n = 3). Repeat injections of 10 nL DHK were made every 15 sec for 10 min. This repetitive injection protocol provided a steady state condition which enabled generation of baroreflex function curves (Fig. 3A). Similar repetitive injections of aCSF were performed as time control.

Baroreflex function curves were obtained before (baseline), during, and after (recovery) these repetitive injections of DHK into the NTS (*n* = 6). The baroreflex function curves of RSNA and HR were constructed during the intravenous infusion of phenylephrine HCI (100 *μ*g/mL) and sodium nitroprusside (100 *μ*g/mL) as previously described (Yamamoto et al. 2013). The rate of infusion (range between 0.5 and 5 mL/h) was adjusted to produce continuous changes in AP. The order delivery of phenylephrine and nitroprusside was randomized.

The values for RSNA and HR during the baroreflex function curves were averaged into 5‐mmHg bins of MAP. We calculated the response ranges of RSNA and HR to changes in MAP (between maximum and minimum RSNA and HR in the reflex function curves).

### Statistical analysis

**Figure 2:**
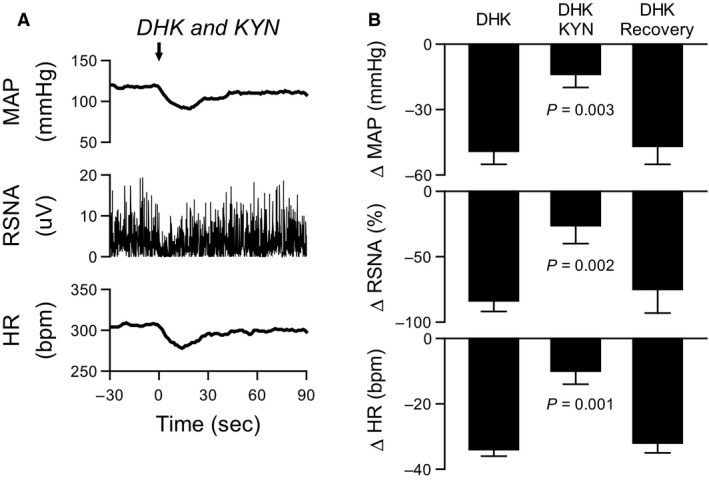
Inhibition of GLT‐1 in the subpostremal NTS decreased MAP, RSNA, and HR by activation of ionotropic glutamate receptors. (A) Typical recordings of MAP, RSNA, and HR in response to the DHK injection during application of kynurenic acid (KYN, glutamate non‐NMDA receptor antagonist) to surface of hindbrain. An arrow represents point of DHK injection. (B) KYN attenuated MAP, RSNA and HR responses to NTS injections of DHK. The DHK response returned to pre‐KYN levels within 30 min. *P* values compared with DHK alone.

**Figure 3:**
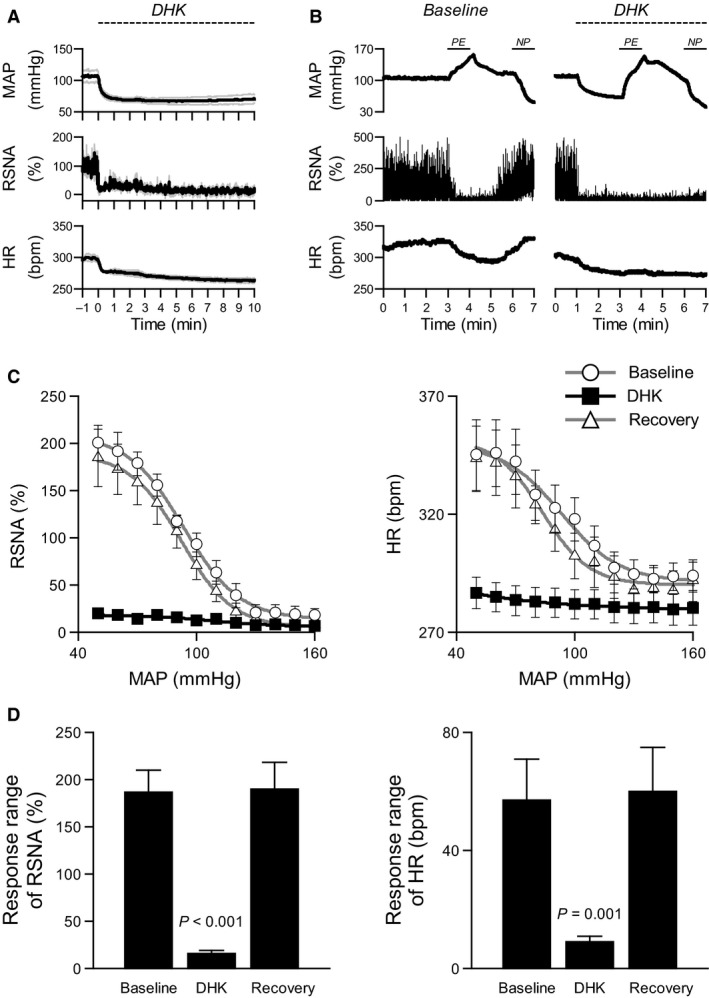
GLT1 in the subpostremal NTS is important to maintain baroreflex control of RSNA and HR. The dashed lines represent period of repetitive DHK injections in A and B. (A) Decreases in MAP, RSNA, and HR maintained during the DHK repetitive injection for 10 min. The solid line indicates the mean value and the gray line represents the mean ± SE. (B) The responses of RSNA and HR to changes in MAP during phenylephrine (PE) and nitroprusside (NP) infusions were attenuated during the inhibition of GLT1. (C) The DHK injections into the NTS eliminated the relationship between MAP and RSNA or HR. The baroreflex curves returned to pre‐DHK levels within 30 min. (D) Baroreflex response ranges of RSNA and HR (between maximum and minimum RSNA or HR in the reflex function curves in Figure 3C) were reduced during inhibition of GLT1. The baroreflex response ranges returned to pre‐DHK levels within 30 min. *P* values compared to baseline.

All data are presented as mean ± SE. MAP, RSNA, and HR responses to the aCSF, TBOA and DHK injections (Fig. 1C) were tested by one‐way ANOVA. The effects of KYN on the DHK responses (Fig. 2B) and the effects of DHK on response range of RSNA and HR in the baroreflex function curves (Fig. 3D) were tested by one‐way ANOVA with repeated measurements. In the case of a significant F value, a post hoc test with Bonferroni method identified significant differences among mean values. Differences were considered significant when *P* < 0.05.

## Results

Figure 1 shows the effect of microinjection of aCSF, TBOA, or DHK into the subpostremal NTS on MAP, RSNA, and HR. Figure 1A illustrates typical recordings of MAP, RSNA, and HR in response to the TBOA and DHK injections. Figure 1B shows typical injection site in subpostremal NTS. Prior to the injections, the baseline value of MAP was 110 ± 2 mmHg, RSNA was 100 ± 0% and HR was 340 ± 8 bpm. The baseline values between aCSF, TBOA, and DHK injections did not differ except when comparing HR in aCSF vs TBOA (*P* = 0.03). The TBOA and DHK injections significantly decreased MAP, RSNA, and HR compared with the responses to aCSF injection (Fig. 1C). The reductions in MAP, RSNA, and HR did not differ between TBOA and DHK injections.

Figure 2 shows the effect of microinjection of DHK into the subpostremal NTS on MAP, RSNA, and HR in the presence of KYN. Figure 2A illustrates typical recordings of MAP, RSNA, and HR in response to the DHK injection in presence of KYN. Topical application of the non‐selective ionotropic glutamate receptor antagonist KYN (5 mmol/L) to the dorsal surface of the brainstem increased baseline MAP by 17 ± 1 mmHg and RSNA by 19 ± 5% (*P* < 0.05). Baseline HR did not change. The KYN application significantly attenuated the responses to NTS injections of DHK on MAP, RSNA, and HR (Fig. 2B). The DHK responses had returned to control levels within 30 min after KYN application (Fig. 2B).

Figure 3A illustrates changes in MAP, RSNA, and HR in response to DHK repetitive injections into the subpostremal NTS for 10 min. Decreases in MAP, RSNA, and HR were maintained during the 10 min DHK injections, indicating that repetitive injection of DHK into the NTS was effective for at least 10 min. Repeated injections of aCSF did not change MAP, RSNA, and HR during a similar 10 min injection.

Figure 3B illustrates MAP, RSNA, and HR responses during pharmacological manipulation of MAP during DHK repetitive injection into the NTS. RSNA and HR decreased in response to the increases in MAP induced by the phenylephrine infusion and increased in response to the decreases in MAP induced by the nitroprusside infusion in baseline conditions (Fig. 3B). The responses of RSNA and HR to similar changes in MAP by the phenylephrine and nitroprusside infusions were attenuated during the DHK injections.

Figure 3C and D show baroreflex curves of RSNA and HR (C) and response ranges of RSNA and HR (D). The DHK injections into the NTS attenuated baroreflex curves of RSNA and HR (Fig. 3C), and it significantly reduced baroreflex response ranges of RSNA by 91% and HR by 83% (Fig. 3D). Baroreflex curves returned to pre‐DHK injection levels by 30 min after the end of the DHK injections (Fig. 3D).

## Discussion

The present study demonstrated that inhibition of astrocytic glutamate transporter GLT‐1 in the subpostremal NTS decreased MAP, RSNA, and HR by activation of ionotropic glutamate receptors. This finding confirms the previous report that decreases in MAP, SNA, and HR induced by inhibition of GLT1 in NTS are mediated, at least in part, by ionotropic glutamate receptors (Matott et al. 2017). The key new finding of the present study is that the activation of glutamate receptors induced by inhibition of GLT1 dramatically reduced baroreflex response ranges of RSNA and HR. The response range is range over which the baroreflex can control SNA and HR to compensate for changes in blood pressure. These results support our hypothesis that inhibition of GLT1 in the NTS attenuates baroreflex control of SNA and HR.

Astrocytes can regulate neuronal activity and synaptic transmission (Fields and Stevens‐Graham 2002; Pascual et al. 2005; Haydon and Carmignoto 2006; Wang et al. 2006; Schummers et al. 2008; Halassa et al. 2009; Gourine et al. 2010; Henneberger et al. 2010; Panatier et al. 2011). Astrocytes within the NTS possess calcium‐permeable AMPA receptors which can be activated by vagal afferent stimulation (McDougal et al. 2011); however, the functional significance of vagal afferent stimulated calcium influx into NTS astrocytes has yet to be determined. The present study provides new information that glutamate transporter on astrocytes in the NTS can modulate arterial baroreceptor reflexes.

We used DHK to inhibit the glutamate transporter on astrocytes, because DHK is selective inhibitor for GLT1 (excitatory amino‐acid transporters; EAAT2) (Arriza et al. 1994; Wang et al. 1998). Although the main glutamate transporter expressed by astrocytes in the NTS is GLT1 (Chounlamountry and Kessler 2011), function in other glutamate transporters such as glutamate/aspartate transporter (GLAST; EAAT1) on astrocytes and excitatory amino acid carrier 1 (EAAC1; EAAT3) on neurons were intact in the NTS. However, it is unlikely that EAAC1 in NTS plays a major role in glutamate clearance (Chounlamountry et al. 2016). The inhibition of GLT1 in the present study dramatically decreased MAP, RSNA, and HR (Fig. 1C) and the decrease in MAP, RSNA, and HR were similar as those induced by TBOA (inhibitor of all subtypes of the transporters) injection. These responses induced by TBOA and DHK injection into the NTS were consistent with recent studies by Matott et al. (2016, 2017). These results provide information that GLT1 in NTS plays an important role in clearing synaptically released glutamate, even at normal levels of blood pressure.

Selective blockade of GLT1 with DHK in the subpostremal NTS decreased MAP, RSNA and HR (Fig. 1C). Application of ionotropic glutamate receptor antagonist KYN attenuated the reductions in MAP, RSNA and HR induced by NTS injections of DHK (Fig. 2B). The responses were not completely blocked by KYN consistent with a previous study (Matott et al. 2017), suggesting that other mechanisms may contribute to the reductions (e.g., activation of metabotropic glutamate receptors). These results indicate that inhibition of GLT1 decreased MAP, RSNA, and HR by activating glutamatergic receptor on neurons due to accumulation of glutamate in the extracellular space in the NTS (Matott et al. 2017). The accumulated glutamate induced a sustained activation of baroreflex pathway in the NTS. As a result, baroreflex control of RSNA and HR was virtually abolished (Fig 3C and D). These findings suggest that glutamate transporter on astrocytes in the NTS is important to maintain baroreflex control of RSNA and HR.

Although DHK selectively blocks GLT1 over other glutamate transporters (Pines et al. 1992; Arriza et al. 1994), a previous study have shown that DHK slightly but directly activates glutamate receptors (Maki et al. 1994). This raises the possibility that DHK‐induced effects on cardiovascular regulation observed in this study could be caused by such a direct action of DHK on glutamate receptors. However, we consider this unlikely for two reasons. First, DHK injection evoked the similar responses with those induced by TBOA injection that has no discernible activity at glutamate receptors (Shimamoto et al. 2004). Second, DHK has been shown to cause accumulation of extracellular glutamate in brainstem NTS slices, and inward currents in NTS neurons were attenuated by ionotropic glutamate receptor blockade (Matott et al. 2017). These observations support the interpretation that DHK works via glutamate rather than a direct effect on glutamate receptors.

Because glutamate is a potent neurotoxin, rapid removal of glutamate from the extracellular space is required for the survival and normal function of neurons. Many studies regarding glutamate transporter on astrocytes have focused on their role in neuroprotection against glutamate toxicity in the brain (Rothstein et al. 1996; Rao et al. 2001). Astrocytes play a crucial role to support neurons after ischemia (Rossi et al. 2007). Impaired astrocyte function can amplify neuronal death (Nedergaard and Dirnagl 2005). Therefore, astrocytes are attractive therapeutic targets in stroke (Nedergaard and Dirnagl 2005; Zhao and Rempe 2010; Barreto et al. 2011). In this study, we provided additional information that glutamate transporter on astrocytes may contribute to, not only the neuronal protection, also maintenance of cardiovascular regulation.

Several studies have recently examined astrocytic modulation of NTS neurons and cardiovascular function. Costa et al. (2013) used fluorocitrate to lesion astrocytes, an in situ preparation from juvenile (19–20 day old rats) and found no change in baseline respiratory and thoracic sympathetic chain discharge. However, a subsequent study by this group using an in vitro brain slice preparation (Accorsi‐Mendonca et al. 2013) found that astrocytic release of ATP acts on presynaptic P2x receptors to increase glutamate release. The authors suggested that inhibition of astrocytes might change neuronal excitability or local synaptic activity as possible explanations for why the results of the two studies differed. Matott et al. (Matott et al. 2016, 2017) found that NTS injections of TBOA or DHK evoked depressor responses similar to those reported here. The authors also found that NTS injections of DHK enhanced arterial chemoreflex pressor responses (Matott et al. 2017), which taken with our observation of GLT1 modulation of baroreflexes, suggests that glial uptake of glutamate is likely to modulate most visceral afferent processes within NTS.

Our result showed that DHK injection into the NTS substantially reduced the response ranges of RSNA and HR in baroreflex stimulus‐response curves. The reduced response ranges were mainly attributed to a decrease in maximum RSNA and HR (Fig. 3C), suggesting a possibility that the sympathoinhibition by DHK in this experiment was near maximal at any MAP level. Thus, it might be not possible to respond to any additional stimulus. For example, activation of baroreflex pathway is a powerful inhibitor of sympathoexcitation induced by somatic afferent activation (Yamamoto et al. 2005). However, the elevated glutamate levels induced by DHK might simply induce a parallel downward shift in the baroreflex stimulus‐response curves. Because of the shift in threshold, the response ranges of RSNA and HR could be reduced (Yamamoto et al. 2005). Future studies will be needed to clarify the effect of a lower concentration of DHK that produces relatively small changes in SNA and AP on baroreflex stimulus‐response curves. As another caveat, we unilaterally injected DHK into the NTS; therefore the baroreflex pathway in the contralateral NTS side was intact and might buffer the DHK responses. This baroreflex closed‐loop condition could confound the data interpretation. In addition, baroreflex gain for SNA depends on the velocity of changes in AP (Yamamoto et al. 2008; Kawada et al. 2011). Analysis of the static and dynamic characteristics of the baroreflex under open‐loop conditions will provide additional insights into the relationship between glial glutamate transporter in NTS and baroreflex function.

In conclusion, the present results indicate that inhibition of astrocytic glutamate transporter GLT‐1 in the subpostremal NTS decreased MAP, RSNA, and HR by activation of ionotropic glutamate receptors in baroreflex pathway. As a result, baroreflex control of RSNA and HR was dramatically attenuated. These findings suggest that astrocytic glutamate transporter in the NTS plays an important role in the maintenance and regulation of cardiovascular function.

## Conflict of Interest

No conflicts of interest, financial or otherwise, are declared by the authors.
